# Draft Genome Sequence of Type Strain *Lysinibacillus xylanilyticus* DSM 23493^T^

**DOI:** 10.1128/genomeA.01037-15

**Published:** 2015-09-10

**Authors:** Guo-hong Liu, Bo Liu, Jie-ping Wang, Jian-Mei Che, Xue-fang Zheng, Qian-Qian Chen, Ci-bin Ge

**Affiliations:** Agricultural Bio-Resources Research Institute, Fujian Academy of Agricultural Sciences, Fujian, China

## Abstract

*Lysinibacillus xylanilyticus* DSM 23493^T^ is a Gram-positive, spore-forming bacterium. Here, we report the 5.22-Mb genome sequence of *Lysinibacillus xylanilyticus* DSM 23493^T^, which will accelerate the application of degrading xylan and provide useful information for genomic taxonomy and phylogenomics of *Bacillus*-like bacteria.

## GENOME ANNOUNCEMENT

In 2007, the genus *Lysinibacillus* was proposed by Ahmed et al. (1) by the description of one novel species and the reclassification of two *Bacillus* species (*Bacillus sphaericus* and *Bacillus fusiformis*) on the basis of a polyphasic taxonomic study, and especially with regard to characteristics such as cell-wall peptidoglycan structure. Since then, more and more novel isolates have been assigned to *Lysinibacillus*. The species classification was mainly based on common features in physiology and phenotype, such as Gram-positive, spore-forming, rod-shaped, motile, presence of the Lys–Asp type of peptidoglycan in the cell wall, the main fatty acids as iso-C_15:0_, and the predominant menaquinones as MK-7 (2).

The type strain *Lysinibacillus xylanilyticus* DSM 23493^T^ proposed by Lee et al. (3) was isolated from forest humus collected from Gyeryong Mountain, Daejeon, Korea, which had the ability of degrading xylan. Here, we present a summary classification and a set of features for *Lysinibacillus xylanilyticus* DSM 23493^T^, together with the description of the genomic sequencing and annotation, in order to improve the understanding of the molecular basis for its ability to degrade xylan.

The genome sequencing of *Lysinibacillus xylanilyticus* DSM 23493^T^ was performed via the Illumina HiSeq 2500 system. Two DNA libraries with insert sizes of 500 and 5,000 bp were constructed and sequenced using the 2 × 150-bp paired-end sequencing strategy. The genome coverage was approximately 150-fold. The reads were assembled via the SOAPdenovo software version 1.05 (4), using a key parameter K setting at 31. Through the data assembly, 13 scaffolds with a total length of 5,221,635 bp were obtained, and the scaffold *N*_50_ was 490,472 bp. The average length of the scaffolds was 401,664 bp, and the longest and shortest scaffolds were 2,306,048 bp and 5390 bp, respectively.

The annotation of the genome was performed using the NCBI Prokaryotic Genomes Automatic Annotation Pipeline (PGAAP) (http://www.ncbi.nlm.nih.gov/genomes/static/Pipeline.html) utilizing GeneMark, Glimmer, and tRNAscan-SE tools (5). A total of 4,933 genes were predicted, including 4,630 coding sequences (CDS), 185 pseudogenes, 99 tRNAs, 18 rRNA genes, and 59 frameshifted genes. The average DNA G+C content was 36.7%, with a slight difference to the value 37.2 mol% acquired by HPLC determination (3).

### Nucleotide sequence accession numbers. 

This whole-genome shotgun project has been deposited at DDBJ/EMBL/GenBank under the accession number LFXJ00000000. The version described in this paper is the first version, LFXJ01000000.
